# Supplementary material for: Tepsin and AP4 mediate transport from the trans-Golgi to the plant-like vacuole in toxoplasma
Source: J Cell Biol. 2025 Oct 13;224(12):e202312109. doi: 10.1083/jcb.202312109 (PMC12517565; doi:10.1083/jcb.202312109)
Supplement: Data S1 — shows maximum likelihood phylogenetic analyses trees constructed using IQ-TREE 2 for apicomplexan adaptor complexes AP1, AP4 subunits (A–D), pan-eukaryotic tepsin (E), and pan-eukaryotic AP1-4 subunits (F–I). [file jcb_202312109_datas1.pdf]

A

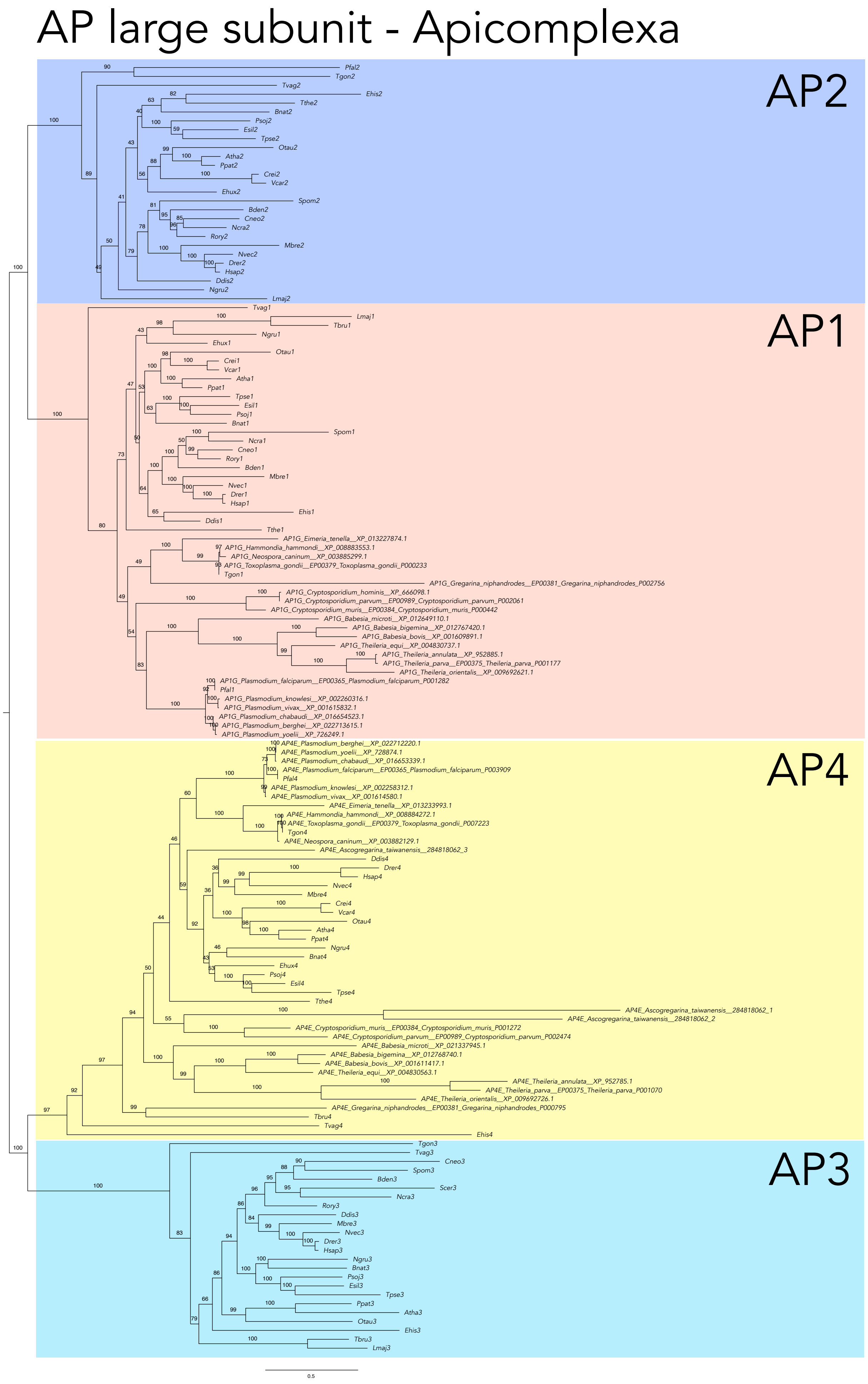
AP large subunit - Apicomplexa
AP2
Pfal2
Tgon2
Tvag2
Ehis2
Tthe2
Bnat2
Psoj2
Esil2
Tpse2
Otau2
Atha2
Ppat2
Crei2
Vcar2
Ehux2
Spom2
Bden2
Cneo2
Ncra2
Rory2
Mbre2
Nvec2
Drer2
Hsap2
Ddis2
Ngru2
Lmaj2
AP1
Tvag1
Lmaj1
Tbru1
Ngru1
Ehux1
Otau1
Crei1
Vcar1
Atha1
Ppat1
Tpse1
Esil1
Psoj1
Bnat1
Spom1
Ncra1
Cneo1
Rory1
Bden1
Mbre1
Nvec1
Drer1
Hsap1
Ehis1
Ddis1
Tthe1
AP1G_Eimeria_tenella__XP_013227874.1
AP1G_Hammondia_hammondi__XP_008883553.1
AP1G_Neospora_caninum__XP_003885299.1
AP1G_Toxoplasma_gondii__EP00379_Toxoplasma_gondii_P000233
Tgon1
AP1G_Gregarina_niphandrodes__EP00381_Gregarina_niphandrodes_P002756
AP1G_Cryptosporidium_hominis__XP_666098.1
AP1G_Cryptosporidium_parvum__EP00989_Cryptosporidium_parvum_P002061
AP1G_Cryptosporidium_muris__EP00384_Cryptosporidium_muris_P000442
AP1G_Babesia_microti__XP_012649110.1
AP1G_Babesia_bigemina__XP_012767420.1
AP1G_Babesia_bovis__XP_001609891.1
AP1G_Theileria_equi__XP_004830737.1
AP1G_Theileria_annulata__XP_952885.1
AP1G_Theileria_parva__EP00375_Theileria_parva_P001177
AP1G_Theileria_orientalis__XP_009692621.1
AP1G_Plasmodium_falciparum__EP00365_Plasmodium_falciparum_P001282
Pfal1
AP1G_Plasmodium_knowlesi__XP_002260316.1
AP1G_Plasmodium_vivax__XP_001615832.1
AP1G_Plasmodium_chabaudi__XP_016654523.1
AP1G_Plasmodium_berghei__XP_022713615.1
AP1G_Plasmodium_yoelii__XP_726249.1
AP4
AP4E_Plasmodium_berghei__XP_022712220.1
AP4E_Plasmodium_yoelii__XP_728874.1
AP4E_Plasmodium_chabaudi__XP_016653339.1
AP4E_Plasmodium_falciparum__EP00365_Plasmodium_falciparum_P003909
Pfal4
AP4E_Plasmodium_knowlesi__XP_002258312.1
AP4E_Plasmodium_vivax__XP_001614580.1
AP4E_Eimeria_tenella__XP_013233993.1
AP4E_Hammondia_hammondi__XP_008884272.1
AP4E_Toxoplasma_gondii__EP00379_Toxoplasma_gondii_P007223
Tgon4
AP4E_Neospora_caninum__XP_003882129.1
AP4E_Ascogregarina_taiwanensis__284818062_3
Ddis4
Drer4
Hsap4
Nvec4
Mbre4
Crei4
Vcar4
Otau4
Atha4
Ppat4
Ngru4
Bnat4
Ehux4
Psoj4
Esil4
Tpse4
Tthe4
AP4E_Ascogregarina_taiwanensis__284818062_1
AP4E_Ascogregarina_taiwanensis__284818062_2
AP4E_Cryptosporidium_muris__EP00384_Cryptosporidium_muris_P001272
AP4E_Cryptosporidium_parvum__EP00989_Cryptosporidium_parvum_P002474
AP4E_Babesia_microti__XP_021337945.1
AP4E_Babesia_bigemina__XP_012768740.1
AP4E_Babesia_bovis__XP_001611417.1
AP4E_Theileria_equi__XP_004830563.1
AP4E_Theileria_annulata__XP_952785.1
AP4E_Theileria_parva__EP00375_Theileria_parva_P001070
AP4E_Theileria_orientalis__XP_009692726.1
AP4E_Gregarina_niphandrodes__EP00381_Gregarina_niphandrodes_P000795
Tbru4
Tvag4
Ehis4
AP3
Tgon3
Tvag3
Cneo3
Spom3
Bden3
Scer3
Ncra3
Rory3
Ddis3
Mbre3
Nvec3
Drer3
Hsap3
Ngru3
Bnat3
Psoj3
Esil3
Tpse3
Ppat3
Atha3
Otau3
Ehis3
Tbru3
Lmaj3
0.5

B

AP beta subunit - Apicomplexa

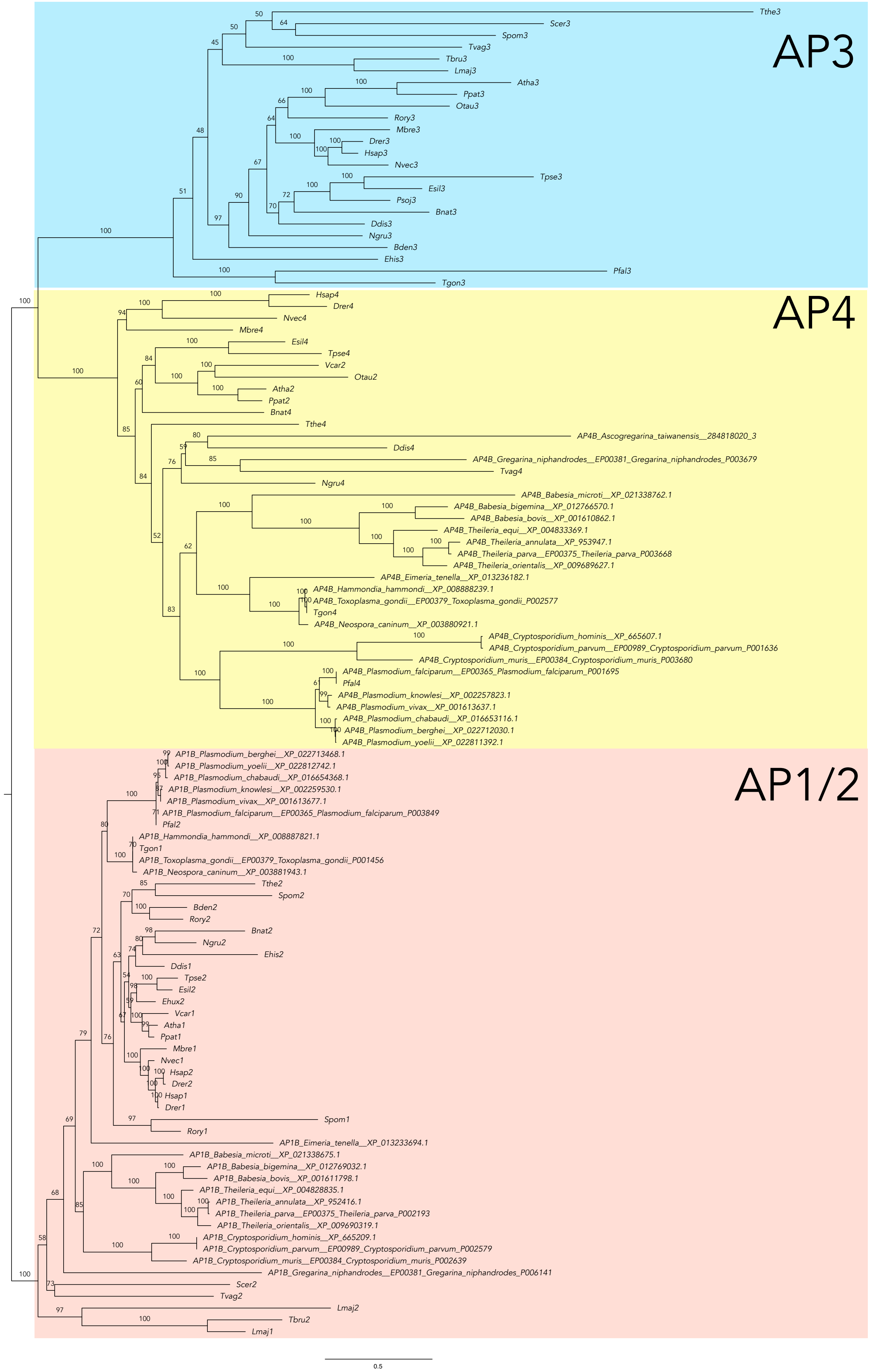

c

AP mu subunit - Apicomplexa

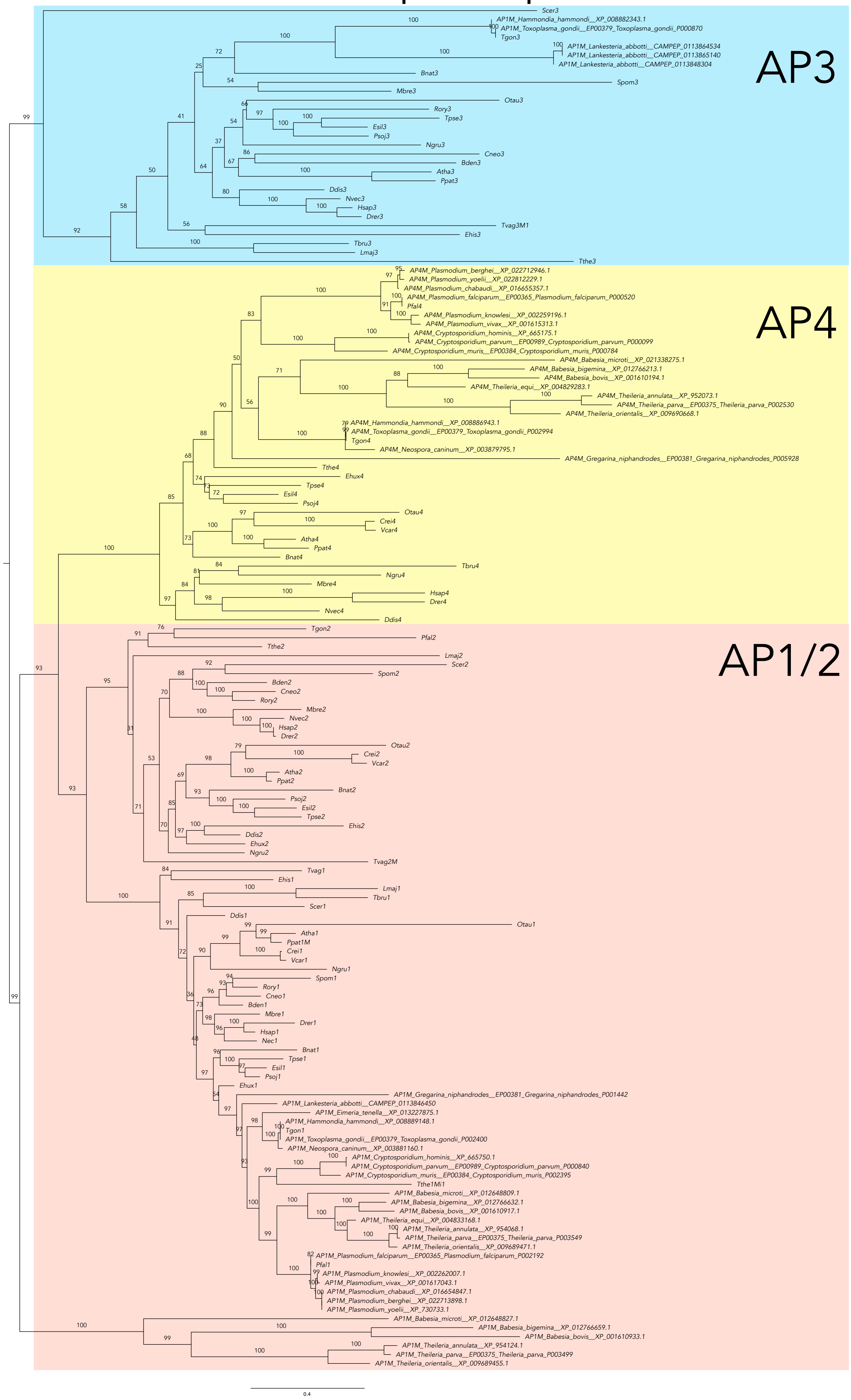

D

AP sigma subunit - Apicomplexa

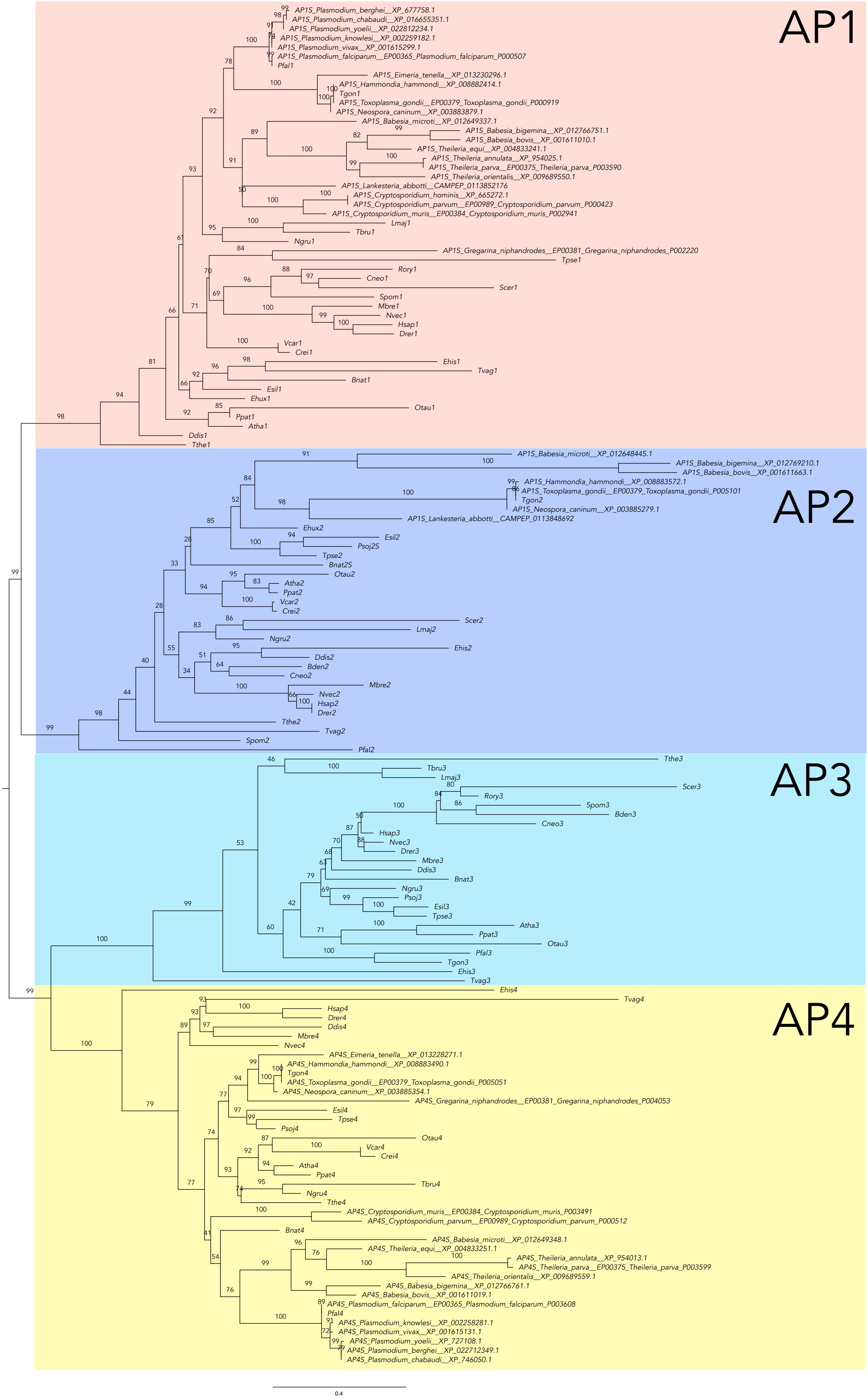

E

Pan-eukaryotic phylogenetic analysis of Tepsin.

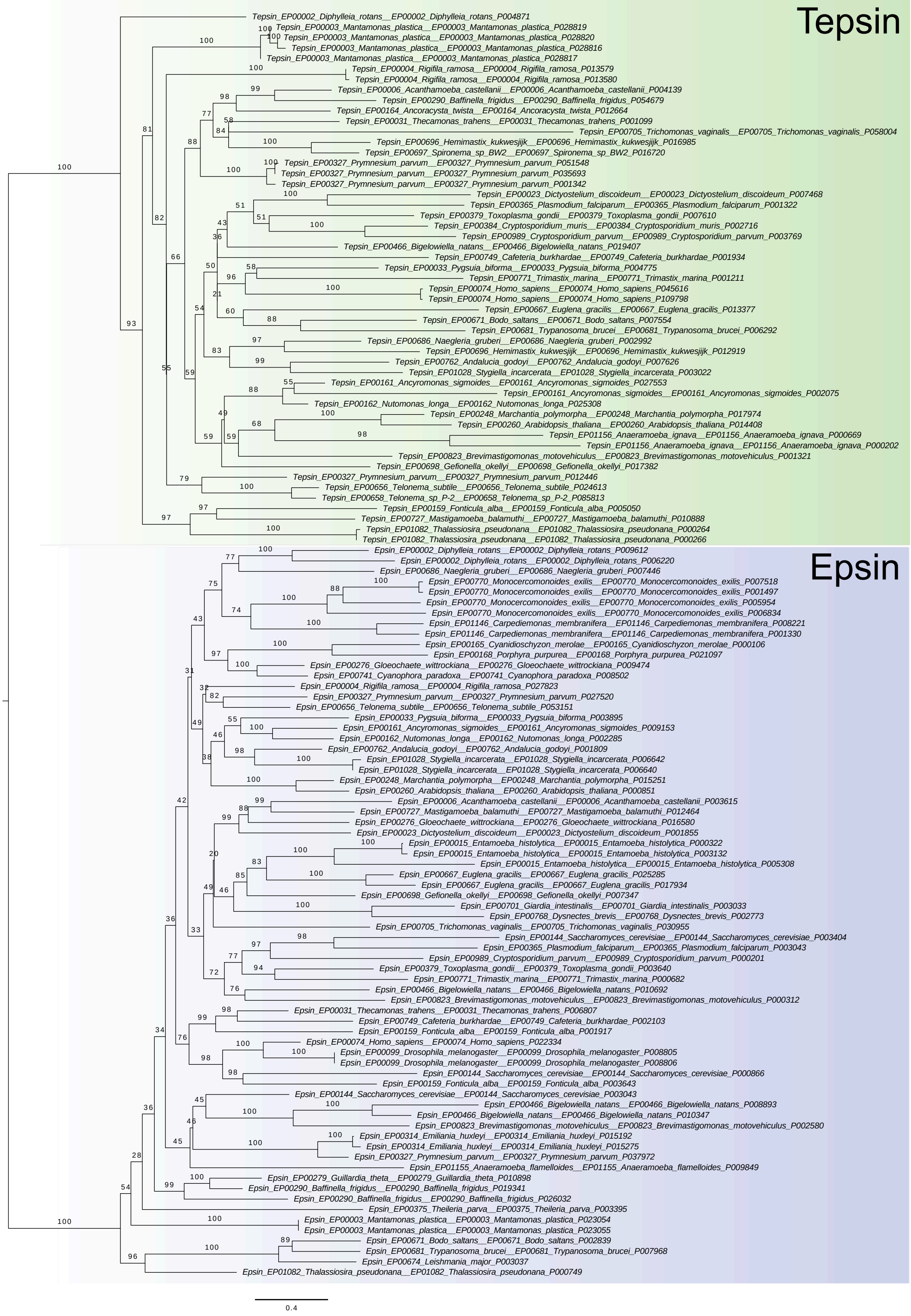

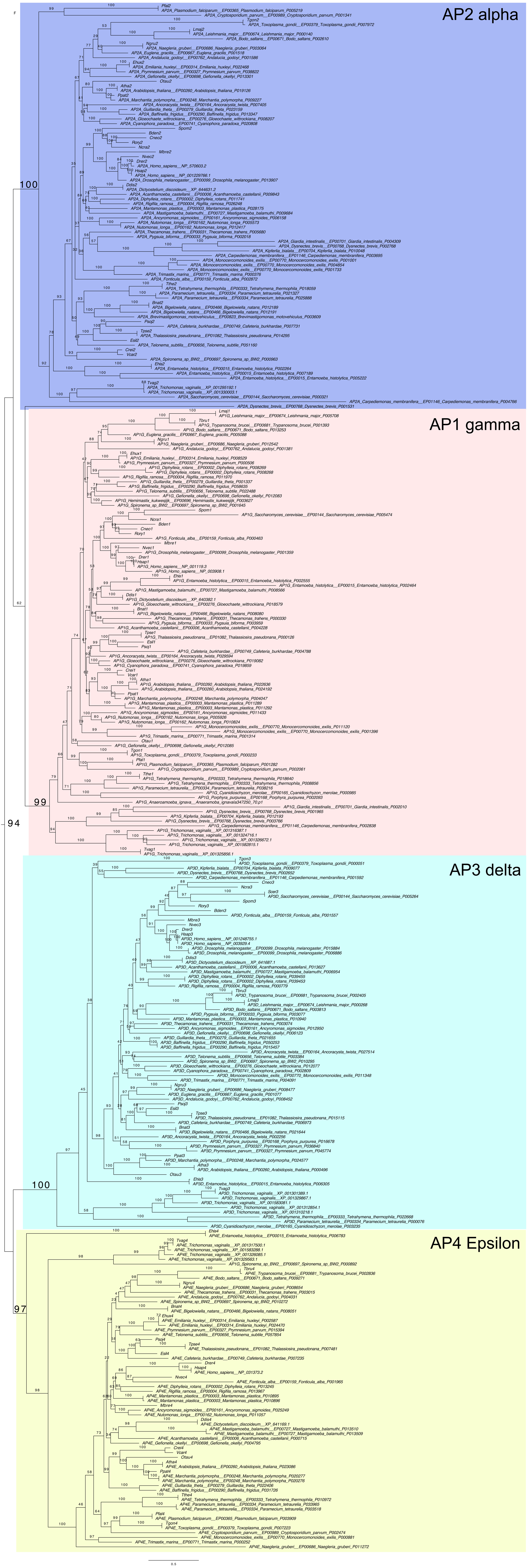

AP2 alpha
AP1 gamma
AP3 delta
AP4 Epsilon
100
99
94
100
97
0.5

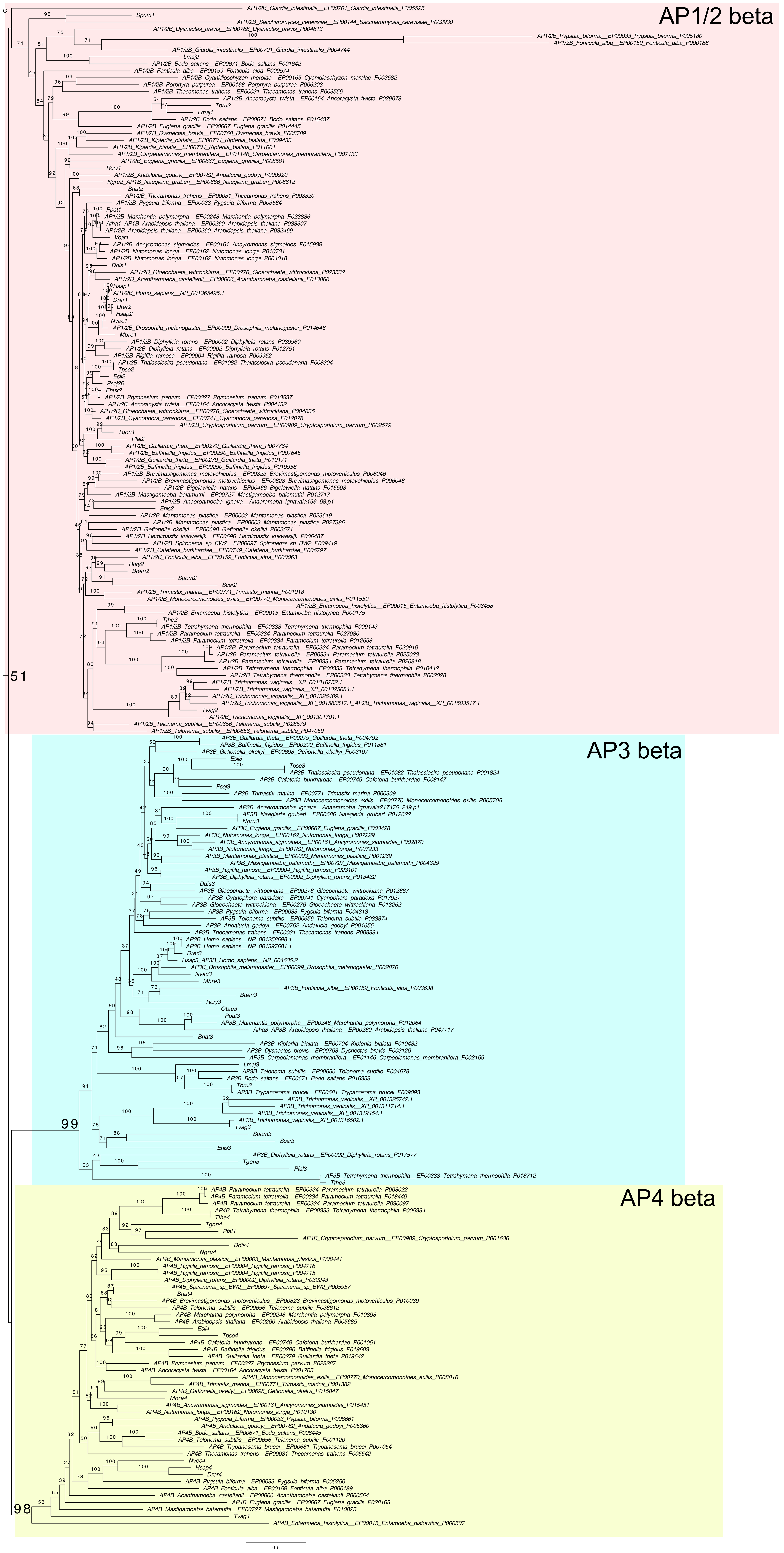

AP1/2 beta
AP3 beta
AP4 beta
0.5

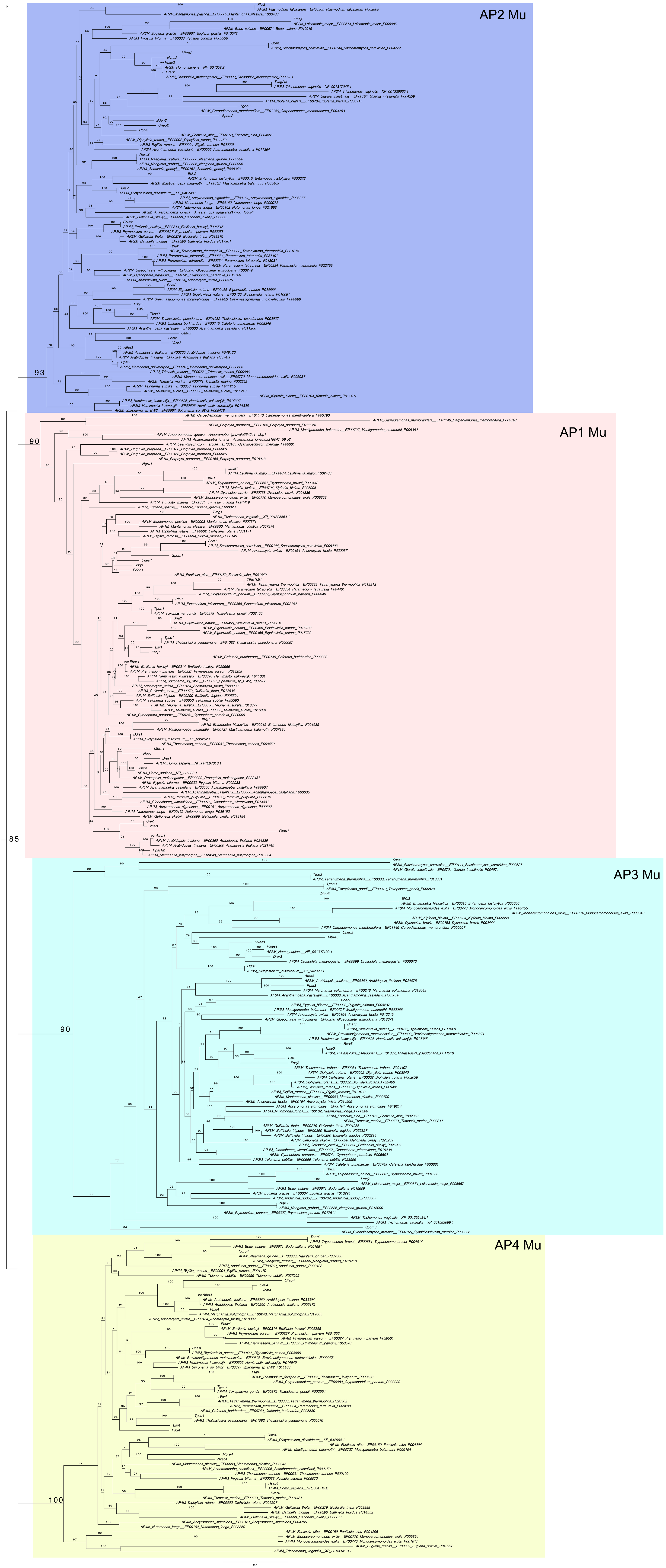
AP2 Mu
AP1 Mu
AP3 Mu
AP4 Mu
93
90
85
90
100

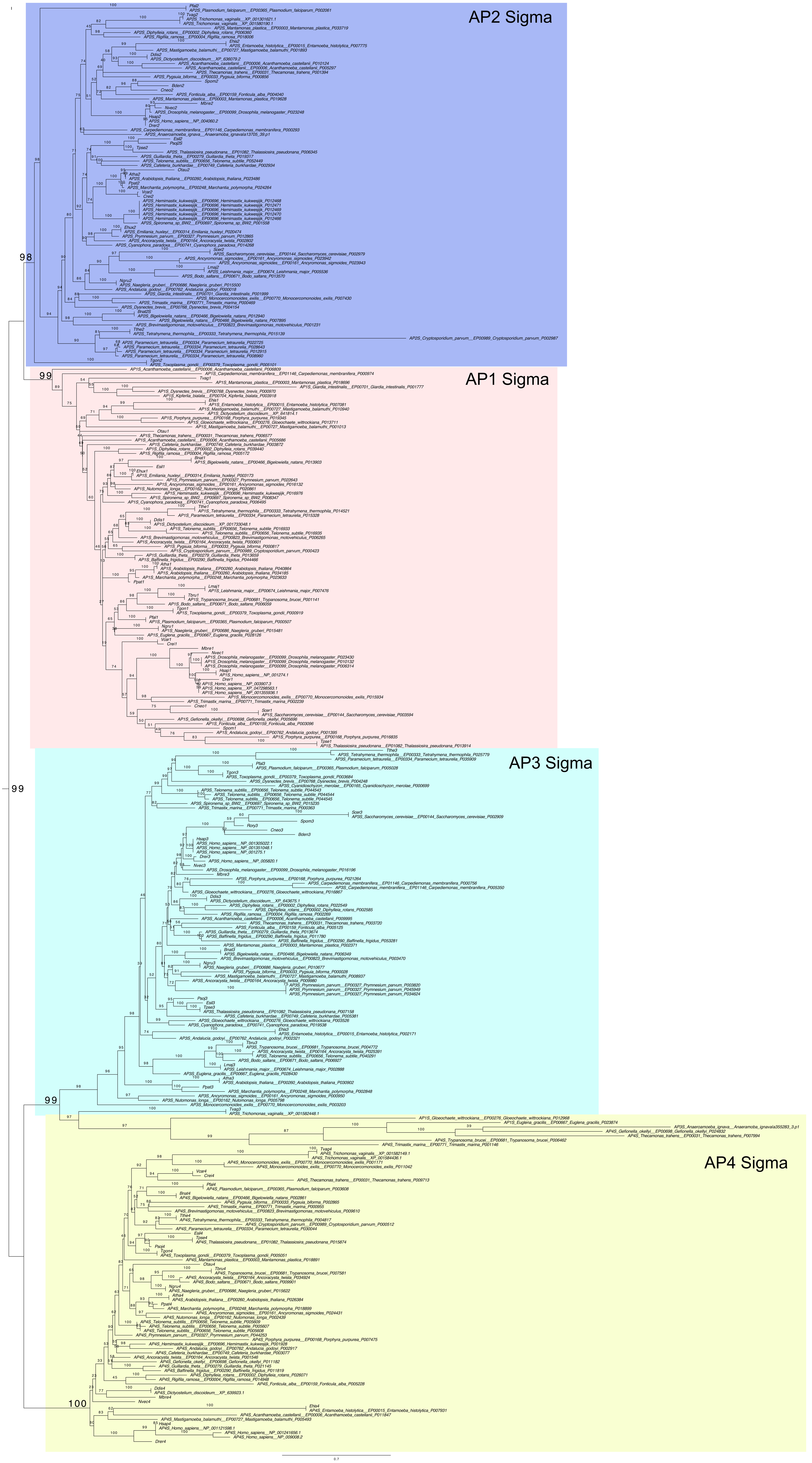
AP2 Sigma
AP1 Sigma
AP3 Sigma
AP4 Sigma
