# Supplementary material for: Tepsin and AP4 mediate transport from the trans-Golgi to the plant-like vacuole in toxoplasma
Source: J Cell Biol. 2025 Oct 13;224(12):e202312109. doi: 10.1083/jcb.202312109 (PMC12517565; doi:10.1083/jcb.202312109)

CoIP Fig.5 700nm channel anti-GFP Gel 1

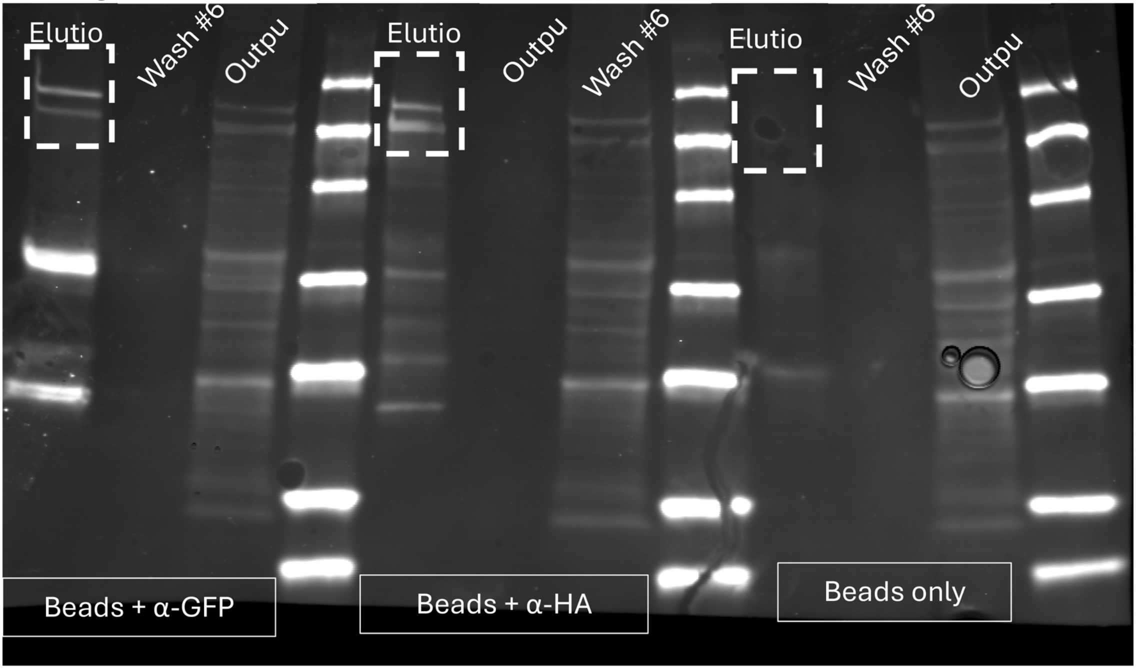

CoIP Fig.5 800nm channel anti-HA Gel 1

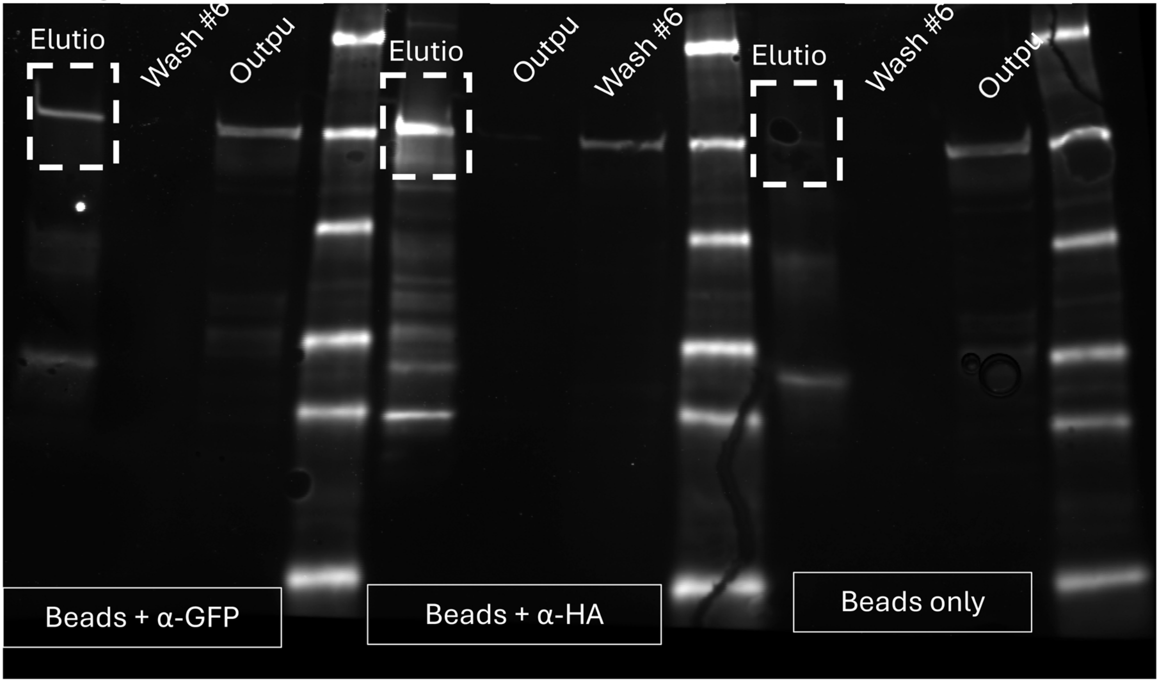

CoIP Fig.5 700nm channel anti-GFP Gel 2

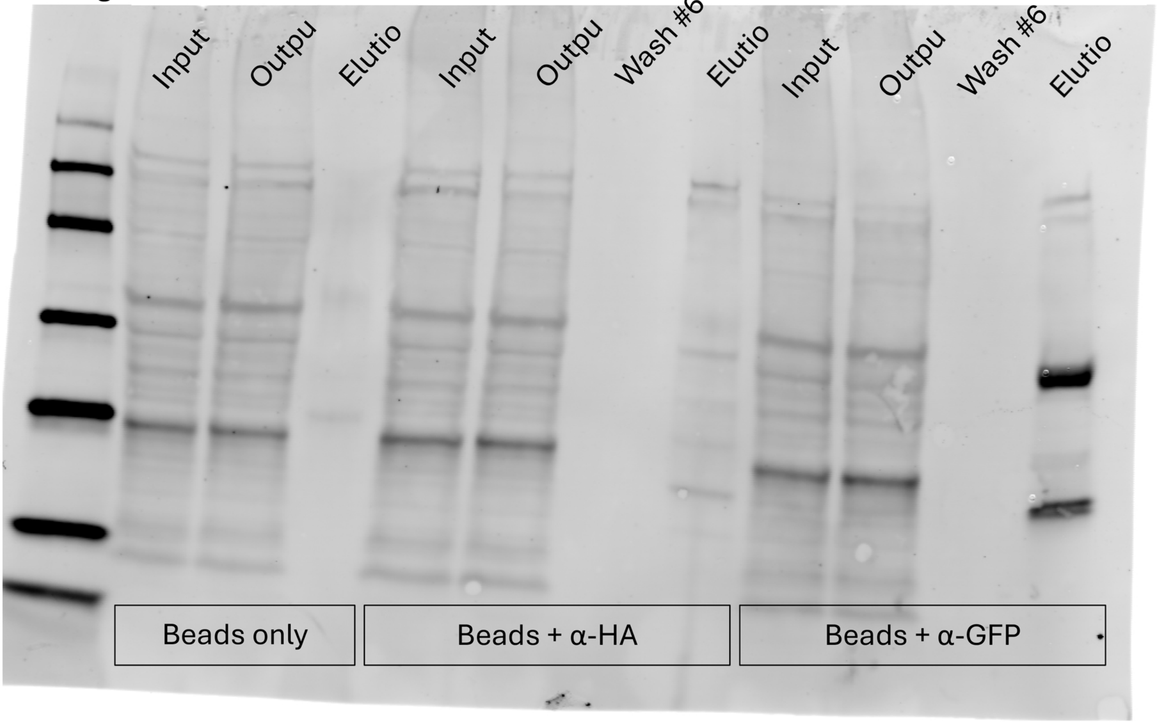

CoIP Fig.5 800nm channel anti-HA Gel 2

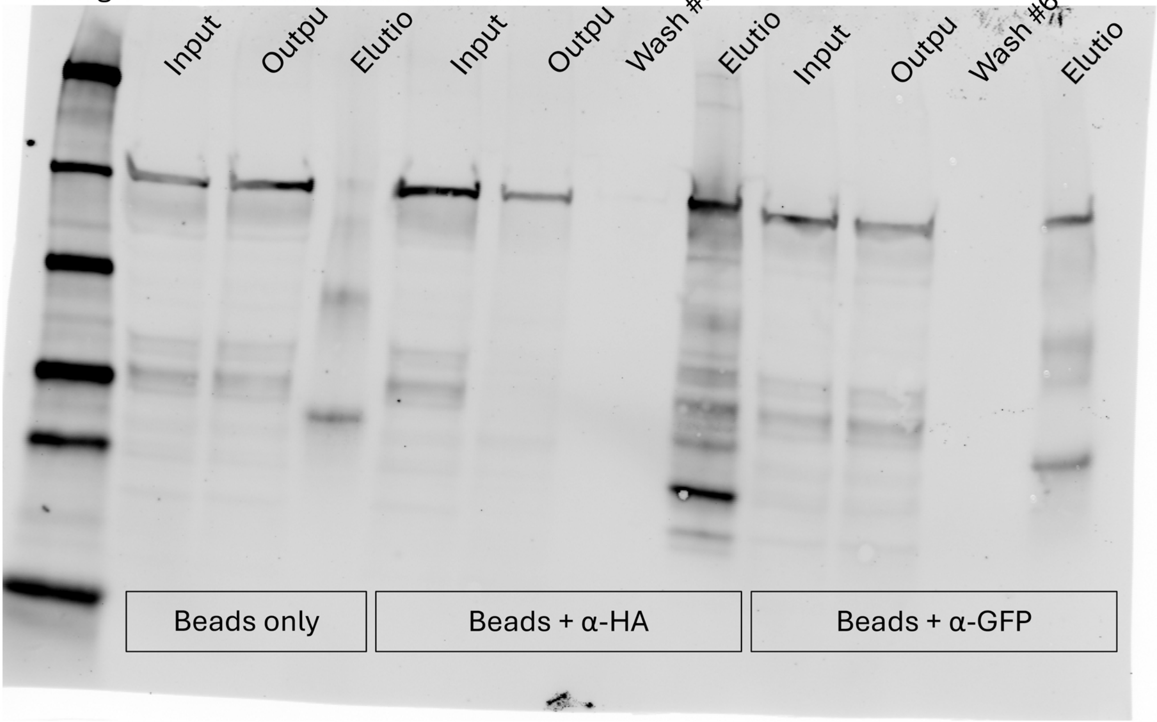

Supplement: SourceData F5 — is the source file for Fig. 5. [file jcb_202312109_sourcedataf5.pdf]
