# Supplementary figures and images for: Tepsin and AP4 mediate transport from the trans-Golgi to the plant-like vacuole in toxoplasma
Source: J Cell Biol. 2025 Oct 13;224(12):e202312109. doi: 10.1083/jcb.202312109 (PMC12517565; doi:10.1083/jcb.202312109)

10000bp  
3000bp  
1000bp  
500bp

301410-mCherry  
301410-sYFP2  
301410-Halo  
301410-TurboID  
WT

-Rapa  
24h  
48h  
72h

+Rapa

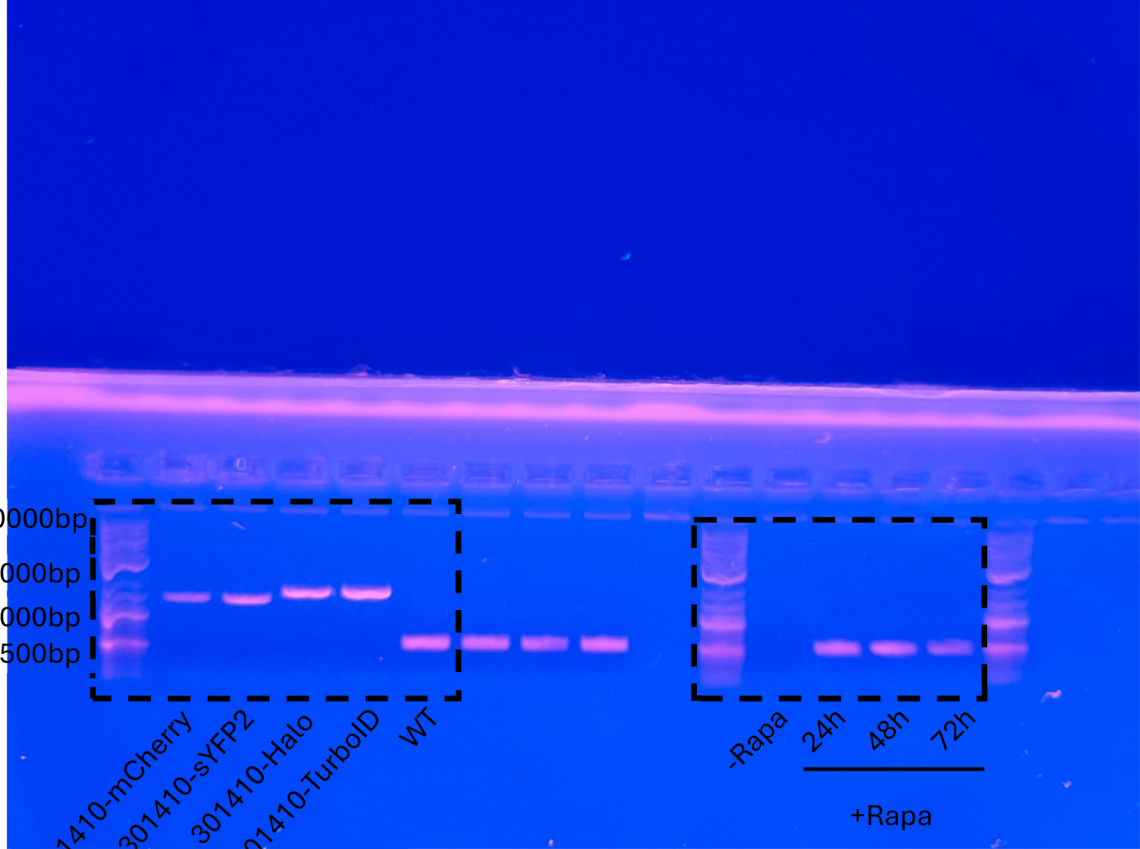

Supplement: SourceData FS1 — is the source file for Fig. S1. [file jcb_202312109_sourcedatafs1.pdf]
